# Ancient oaks reveal rewilding of Mediterranean forests after the Black Death

**DOI:** 10.1073/pnas.2529341123

**Published:** 2026-06-01

**Authors:** Gianluca Piovesan, Michele Baliva, Franco Biondi, Jordan Palli, Lucio Calcagnile, Alessandro Chiarucci, Raffaele Manicone, Gianluca Quarta, Giovanni Quilghini, Antonino Siclari, Charles H. Cannon

**Affiliations:** ^a^ Department of Ecological and Biological Sciences, University of Tuscia, Viterbo 01100, Italy; ^b^ DendroLab, Department of Natural Resources and Environmental Science, University of Nevada, Reno, NV 89557; ^c^ Department of Mathematics and Physics “Ennio De Giorgi”, Centre of Applied Physics, Dating and Diagnostics, University of Salento, Lecce 73100, Italy; ^d^ Department of Biological, Geological, and Environmental Sciences, Alma Mater Studiorum University of Bologna, Bologna 40126, Italy; ^e^Comando Unità Forestali Ambientali ed Agroalimentari, Raggruppamento Carabinieri Biodiversità, Roma 00187, Italy; ^f^Città Metropolitana di Reggio Calabria, Reggio Calabria 89100, Italy; ^g^ Applied Research Center for Tropical Plant Conservation, Xishuangbanna Tropical Botanical Garden (XTBG), Menglun 666303, China

**Keywords:** ancient trees, old-growth, past pandemics, radiocarbon dating, rewilding

## Abstract

In Italy, evergreen holm oaks (*Quercus ilex*) and deciduous sessile oaks (*Quercus petraea*) experienced a synchronized establishment pulse starting at the beginning of 1400s CE, consistent with a release from anthropogenic pressure following demographic collapse associated with the Black Death outbreak (1347 CE) and its following waves until the 17th century. Radiocarbon dating of the oldest-looking individuals of each species revealed similar tree-age distributions across sites located at the extremes of the forest elevational gradient: Montecristo Island (100 to 500 m a.s.l.) and Aspromonte mountain (1,100 to 1,800 m a.s.l.). Montecristo, with its favorable coastal conditions, showed a pronounced post-1400 CE recruitment peak, while the mountainous Aspromonte region, at harsher high elevations, required a more extended and delayed period of recruitment. The extreme longevity of these two unrelated oak populations is enabled by different functional traits and resilience mechanisms, highlighting an ancient rewilding event spurred by human tragedy.

The Mediterranean basin is a repository of millennia-long histories, both cultural and natural, tragic and uplifting. For instance, the collapse of human populations and economic activities following the Black Death pandemic (1347 to 1350 CE) coincided with widespread forest expansion across diverse European landscapes (1). Determining the ages of ancient trees remains a challenging task, and as a result, the demographic structure of old-growth tree populations is still poorly understood (2, 3). Consequently, our knowledge of the biological and ecological mechanisms underlying tree longevity (4, 5) and their relationships with past human civilizations remain largely anecdotal.

Building on recent discoveries of centuries- and millennia-old trees (6, 7), we used radiocarbon dating to investigate the age structure of two broadleaf populations—evergreen holm oak (*Quercus ilex*) on Montecristo Island and deciduous sessile oak (*Quercus petraea* subsp. *austrotyrrhenica*) on the Aspromonte Massif (Fig. 1)—occurring in distinct bioclimatic zones at opposite ends of the Mediterranean forest elevational range.

**Fig. 1.**
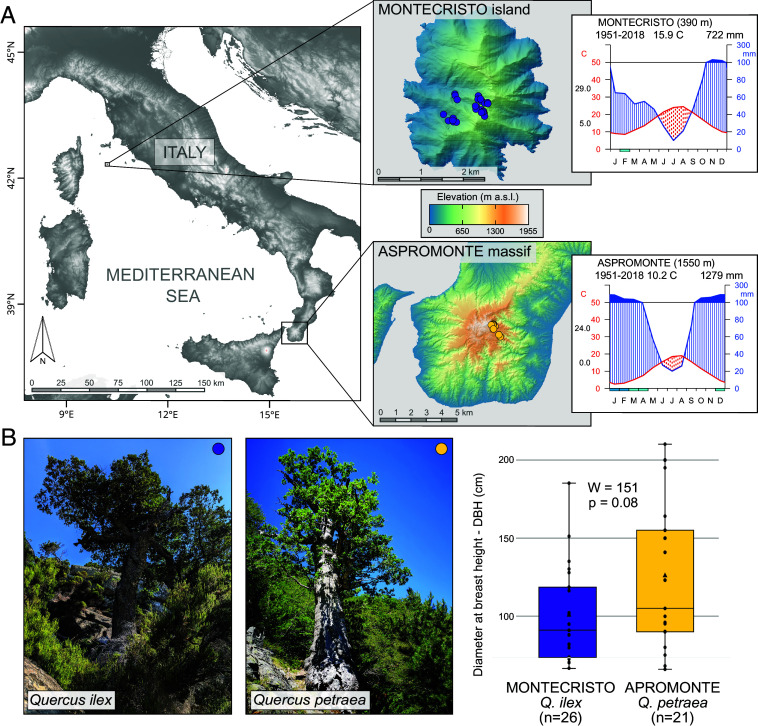
Site description. (*A*) Map of the two sampled stands showing terrain topography and termo-pluviometric diagrams. See *SI Appendix* for details. (*B*) Representative old trees with color-matched boxplots showing diameter distributions of the two stands. Boxplot statistics, based on the Wilcoxon rank-sum test, indicate no significant differences in diameter distribution between the two stands.

## Results and Discussion

**Fig. 2.**
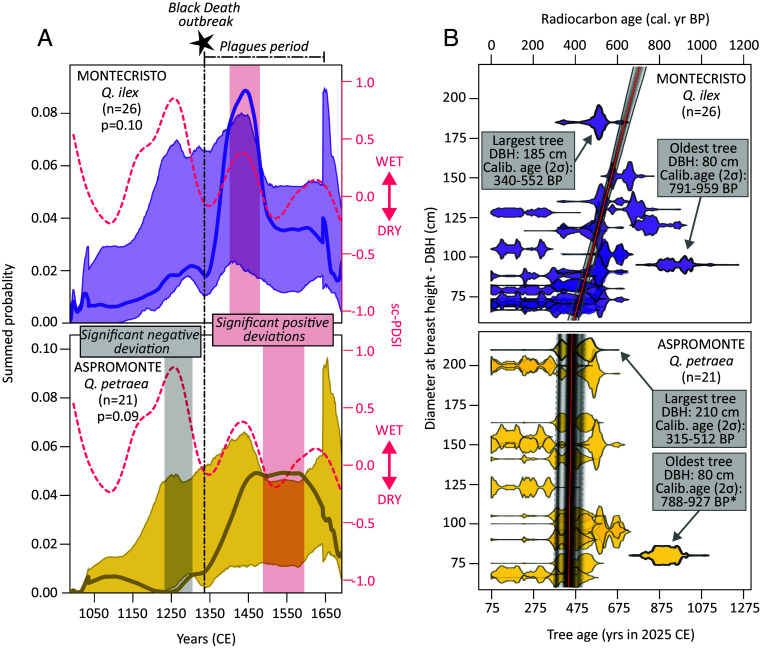
Age distribution models. (*A*) Summed probability distributions (SPD) of radiocarbon dates for Montecristo (purple) and Aspromonte (yellow) oaks. The 95% confidence envelope of the Monte-Carlo simulated SPD (shaded regions) is compared to the observed SPD (solid lines). Red bars denote significant positive deviations above the confidence envelope (recruitment pulses), while the gray bar indicate negative deviations (regeneration failure or mortality). The outbreak of the Black Death (black star) and subsequent plague waves are shown together with a smoothed self-calibrating-PDSI reconstruction (dashed pink line) (8). (*B*) Age-diameter Bayesian model calculated on calibrated radiocarbon dates. The red line indicates the mean modeled age range with associated uncertainty (2σ), represented by the dark shaded area. Ages are shown as calibrated years BP and tree ages in 2025. Gray boxes indicate the radiocarbon age range and DBH of the largest and oldest individuals [the asterisk denotes ages obtained by wiggle-matching (7)]. See *SI Appendix* for details.

A comparative analysis of the age-distribution probability profiles for old oaks at both sites revealed a concentration of establishment dates between 1400 and 1650 CE, a period that coincides with recurring plague waves across Italy and the broader Mediterranean region (Fig. 2*A*). On Montecristo Island, *Q. ilex* displayed a significant positive deviation from the expected summed probability distribution (SPD) between 1407 and 1486 CE, a wave of tree establishment occurring a few decades after the 1347 Black Death outbreak (Fig. 2*A*). Reduced human pressure likely allowed widespread rewilding on the island, aided by a wet phase that promoted forest recovery (Fig. 2*A*) (8). In contrast, the Aspromonte *Q. petraea* population exhibited a significant negative SPD deviation during Medieval times (1237 to 1310 CE) and a more delayed post–Black Death tree recovery. This delay can be attributed to intense human disturbance in the mountain environment during the Medieval period, which degraded the forest ecosystem and postponed sessile oak’s establishment to a later, drier climatic period (Fig. 2*A*). This interpretation is supported by similar establishment peaks observed among subalpine pine populations in Mediterranean mountain regions during the late 15th century (9). While factors such as fire and extreme weather events can strongly influence stand regeneration dynamics, the occurrence of a synchronized pulse of tree recruitment across environments as distant and contrasting as a remote northern Tyrrhenian island and the high mountains of southern Italy argues against natural disturbances being the primary drivers of the observed regeneration pulse. The Black Death pandemic is the most plausible release mechanism for a synchronized tree recruitment pulse across two distant, contrasting Italian oak stands, leaving a demographic legacy in the forest age structure that remains detectable after six centuries. Palynological records further corroborate a post–Black Death rewilding phase affecting the Italian peninsula (10) and other European regions (1).

The SPD peak of *Q. ilex* on Montecristo Island (Fig. 2*A*) suggests that the stand recovered within ~100 y to become the dense forest described in historical accounts from the 16th century (Enrico D’Alberti, Crociera del Violante, 1876; Annali del Museo Civico di Storia Naturale di Genova). Recently, feral goat herbivory has caused a demographic collapse in oak forests, precluding regeneration and leaving only senescent trees (6); goat population control is therefore necessary. In Aspromonte, the delayed and broader significant positive SPD deviations (Fig. 2*A*) are consistent with grazed parkland forests in a mountain environment, where ecosystem rewilding proceeds more slowly due to the harsher climate and shorter growing season, as observed in other southern Italian mountains (9).

The synchronized tree establishment observed ~650 y ago carries important implications for tree biology and applied ecology, including demographic models relevant to the carbon cycle (11). These two oak populations harbor the oldest angiosperm trees in the temperate zone, and this finding underscores the need to study the complex demography of long-lived tree species in natural ecosystems as well as human-modified forest landscapes.

Radiocarbon dating has pushed the recorded maximum longevity of angiosperms trees beyond the 500 to 600 y threshold typically reported in tree-ring studies (12, 13). Among hardwoods, the genus *Quercus* was already known for its exceptional longevity at relatively high elevations (7), but the discovery of holm oaks of near-millennial ages (950 ± 84 y in 2025; Fig. 2*B*) on Montecristo Island extends previous longevity estimates for Mediterranean evergreen broadleaved species by roughly 200 y (13). Mediterranean oaks possess traits that can confer a long life, including dense and durable wood, drought resistance mechanisms, and a capacity for crown regeneration and resprouting (3). Notably, all of the oldest Mediterranean trees occur in harsh, rocky environments where climate variability strongly influences tree establishment, growth, and survival (3).

This study demonstrated that trees of similar ages exhibit a large variation in diameter due to differing growth rates. The oldest individuals displayed below-average growth rates (Fig. 2*B*). Overall, our findings corroborate the observation that the largest trees are not necessarily the oldest, and that ancient individuals constitute only a small fraction of the total old-tree population (2, 3).

Furthermore, the evolutionary advantage of such extended tree longevity warrants consideration. The success of millennium-scale lifespans in two oak species with contrasting functional traits (deciduous vs. evergreen; ring-porous vs. diffuse-porous wood; medium vs. high-density wood) and biogeographic requirements highlights the key role of longevity for life-history strategies, enabling the wide distribution of *Quercus* across diverse temperate environments. This study provides further evidence that tree longevity is the result of the interplay between life history traits and niche environmental interactions, which then control the chance of tree survival (3). Ancient trees provide an invaluable living heritage as a natural archive for reconstructing past environments and a hub of biodiversity and resilience for perpetuating functional forest ecosystems (14, 15). These ecosystems are also capable of recovering once disturbance pressures are removed, providing evidence that land sparing and forest restoration are effective conservation mechanisms.

## Materials and Methods

Wood samples were extracted to balance the need for a reduced number of tree rings per sample with the optimal mass required for accurate radiocarbon determinations. Radiocarbon dating was performed using Accelerator Mass Spectrometry (AMS) followed by calibration to calendar years using the INTCAL20 calibration curve in the OxCal 4.4 software. An age-diameter Bayesian model was fit separately to the two study areas and reconstructed establishment dates of Mediterranean oaks were compared with proxy-derived drought histories. Full details on sampling, radiocarbon, and numerical methods are provided in *SI Appendix*.

## Supplementary Material

Appendix 01 (PDF)

## Data Availability

Excel spreadsheet with raw and calibrated radiocarbon data have been deposited in Open Science Framework (16).
