# Supplementary material for: Ancient oaks reveal rewilding of Mediterranean forests after the Black Death
Source: Proc Natl Acad Sci U S A. 2026 Jun 1;123(24):e2529341123. doi: 10.1073/pnas.2529341123 (PMC13273268; doi:10.1073/pnas.2529341123)
Supplement: Supplementary file 1 — Appendix 01 (PDF) [file pnas.2529341123.sapp.pdf]

Supporting Information for
Ancient oaks reveal rewilding of Mediterranean forests after the Black Death

Gianluca Piovesan[1], Michele Baliva[1], Franco Biondi[2], Jordan Palli[1]*, Lucio Calcagnile[3], Alessandro Chiarucci[4], Raffaele Manicone[5], Gianluca Quarta[3], Giovanni Quilghini[5], Antonino Siclari[6], Charles H. Cannon[7]

* Dr. Jordan Palli
Email: jo.palli@unitus.it

This PDF file includes:

Supporting text
SI References

Supporting Information Text

Extended Methods

Old wood sampling. Wood samples were extracted from selected putative old individuals during two field campaigns, respectively from 2018 to 2022 in Aspromonte National Park (southern Italy) and from 2018 to 2024 in Montecristo Island (Tuscan Archipelago National Park, Italy) (Fig. 1). Sampling in Aspromonte encompassed multiple nearby sites in a high-mountain setting at elevations between 1,100 and 1,800 m a.s.l., within a parkland environment characterized by a gradient of canopy cover, from scattered trees to closed forest. Most sampled trees were located on slopes with N–NE aspect, except for five individuals growing on S–SW aspect. On Montecristo Island, the sampled population consists of scattered individuals growing within a low Mediterranean shrubland on N-NW facing slopes, between 100 and 500 m a.s.l. Sampling operations involved 21 *Quercus petraea* (Matt.) Liebl. subsp. *austrotyrrhenica* Brullo, Guarino & Siracusa trees in Aspromonte, and 26 *Quercus ilex* L. trees on Montecristo. Wood samples were extracted from the innermost and lowest part of the trunk, as close as possible to the root collar and pith. Sample sizes were determined to balance the need for a reduced number of tree rings per sample with the optimal mass required for accurate radiocarbon determinations. In hollowed trunks, the effective sampling of the oldest wood using specialized tools depended on both the recovery of wood fragments adjacent to the pith and the accessibility of the lower trunk section through natural cavities. Additional methods and technical details are described in (1, 2), which also present part of the data used in this paper. All sampled wood fragments exhibited a moderate curvature of tree rings; however, a reliable estimation of pith offset (i.e., the number of missing rings between the innermost sampled annual increment and the pith) could not be achieved. Due to the potential pith offset and the vertical sampling distance from the collar (up to 1 m), in some trees the obtained radiocarbon dates might underestimate true ages by a few decades.

Radiocarbon dating. Radiocarbon dating was performed using Accelerator Mass Spectrometry (AMS) at CEDAD (Centre of Applied Physics, Dating and Diagnostics), University of Salento, Lecce, Italy. Wood samples underwent chemical pre-treatment to eliminate potential contaminants and to convert them into a form suitable for AMS measurement. This involved the standard Acid-Alkali-Acid (AAA) protocol, consisting of sequential treatments with Acid (HCl, 1M, room temperature until pH=1), Alkali (NaOH, 1M, 60°C), and Acid (HCl, 1M, room temperature) (3). The purified cellulose was then sealed in quartz tubes with CuO and silver wool and combusted to CO_2 at 900°C. The extracted CO_2 was cryogenically purified under vacuum and subsequently converted to approximately 1 mg of graphite per sample in dedicated reaction cells using H_2 as a reducing agent and iron powder as a catalyst (4). The resulting graphite was used for measuring the radiocarbon concentration with a 3 MV Tandetron-type AMS system (Mod. 4130HC, HVEE). The $^{14}C/^{12}C$ ratios of the samples were determined by comparing the ion beam currents and radiocarbon counts with those obtained from standard materials of known ^{14}C concentration (IAEA C6 sucrose) (5).
The measured $^{14}C/^{12}C$ isotopic ratios were corrected for mass fractionation using the $\delta^{13}C$ values measured online by AMS and for background contributions to obtain conventional radiocarbon ages based on the radiocarbon decay law. Uncertainty was calculated as the larger value between the error resulting from radiocarbon counting (Poisson statistics) and the standard deviation of ten repeated measurements performed on each sample. The obtained radiocarbon ages were then calibrated to calendar years using the INTCAL20 calibration curve (6) and the OxCal 4.4 software (7). The final uncertainty in the calibrated ages accounts for and is influenced by the shape of the calibration curve, which can result in non-Gaussian, multi-modal distributions of the calibrated time ranges for some samples (8).

Age distribution models. To investigate the age pattern and the demography of old trees within each site, we employed a Monte-Carlo simulation-based tests of the Summed Probability Distribution (SPD) of radiocarbon dates with the R package "*rcarbon*" (9) and an age-diameter Bayesian model with the R package *rbacon* (10).

The Monte-Carlo simulation approach fits the observed SPD to a theoretical growth model to generate an expected SPD and identify local divergence of the observed SPD from the fitted model (9). The time window was constrained by the oldest dated oak tree (980 yrs) and a theoretical assumption of a mean age of 250 BP, calculated averaging the age of old and ancient trees reported in (11) in the case of 1% mortality rate. Analyses were performed independently for each site, and summary statistics together with graphical outputs were produced to evaluate deviations of the observed SPD from the critical envelope that encompasses the middle 95% of the simulated SPDs. Significant deviations indicate periods where the intensity of radiocarbon dates was unexpectedly higher or lower than predicted under the theoretical growth model.
The age-diameter Bayesian model combines radiocarbon dates modelled using a student-t distribution with wide tails and generates an autoregressive semiparametric model with an arbitrary number of subdivisions, a method widely used for constructing chronologies of lake and peat sediments (10). The model was used in this paper to ameliorate the uncertainty in ^{14}C measurements and examine the relationship between radiocarbon-based tree age estimates and tree diameter in the two study stands.

Climate data. Termo-pluviometric (Walter-Lieth) diagrams displayed in Figure 1 were generated using the R package *climatol* (https://www.climatol.eu/). For the Montecristo island, we used version 4 of the CRU TS monthly high-resolution gridded multivariate climate dataset (12). For the Aspromonte massif, we used the E-OBS dataset from the EU-FP6 project UERRA (https://www.uerra.eu) and the Copernicus Climate Change Service from the ECA&D project (https://www.ecad.eu) (13). Reconstructed establishment dates of Mediterranean oaks were compared with the self-calibrated Palmer's Drought Severity Index (sc-PDSI) developed using tree-ring δ18O records from the European Alps (14) over the last 1000 years. The PDSI curve, initially resolved at 5-year intervals, was subsequently smoothed using a cubic spline with a 30-year smoothing window, resulting in an effective temporal resolution of 150 years.

SI References

1. G. Filibeck, *et al.*, Rediscovering Montecristo's treasure: The island's holm oaks reveal exceptional longevity. *Ecology* **104**, e4064 (2023).
2. G. Piovesan, *et al.*, Radiocarbon dating of Aspromonte sessile oaks reveals the oldest dated temperate flowering tree in the world. *Ecology* (2020). https://doi.org/10.1002/ecy.3179.
3. G. Quarta, M. D'Elia, D. Valzano, L. Calcagnile, New Bomb Pulse Radiocarbon Records from Annual Tree Rings in the Northern Hemisphere Temperate Region. *Radiocarbon* **47**, 27–30 (2005).
4. M. D'Elia, *et al.*, Sample preparation and blank values at the AMS radiocarbon facility of the University of Lecce. *Nucl. Instruments Methods Phys. Res. Sect. B Beam Interact. with Mater. Atoms* **223–224**, 278–283 (2004).
5. L. Calcagnile, G. Quarta, M. D'Elia, High-resolution accelerator-based mass spectrometry: precision, accuracy and background. *Appl. Radiat. Isot.* **62**, 623–629 (2005).
6. P. J. Reimer, *et al.*, The IntCal20 Northern Hemisphere Radiocarbon Age Calibration Curve (0–55 cal kBP). *Radiocarbon* **62**, 725–757 (2020).
7. C. B. Ramsey, S. Lee, Recent and Planned Developments of the Program OxCal. *Radiocarbon* **55**, 720–730 (2013).
8. J. Van Der Plicht, C. Bronk Ramsey, T. J. Heaton, E. M. Scott, S. Talamo, Recent Developments in Calibration for Archaeological and Environmental Samples. *Radiocarbon* **62**, 1095–1117 (2020).
9. E. R. Crema, A. Bevan, INFERENCE FROM LARGE SETS OF RADIOCARBON DATES: SOFTWARE AND METHODS. *Radiocarbon* **63**, 23–39 (2021).
10. M. Blaauw, J. A. Christeny, Flexible paleoclimate age-depth models using an autoregressive gamma process. *Bayesian Anal.* **6**, 457–474 (2011).
11. C. H. Cannon, G. Piovesan, S. Munné-Bosch, Old and ancient trees are life history lottery winners and vital evolutionary resources for long-term adaptive capacity. *Nat. Plants 2022 82* **8**, 136–145 (2022).

12. I. Harris, T. J. Osborn, P. Jones, D. Lister, Version 4 of the CRU TS monthly high-resolution gridded multivariate climate dataset. *Sci. Data* **7**, 1–18 (2020).
13. R. C. Cornes, G. van der Schrier, E. J. M. van den Besselaar, P. D. Jones, An Ensemble Version of the E-OBS Temperature and Precipitation Data Sets. *J. Geophys. Res. Atmos.* **123**, 9391–9409 (2018).
14. T. Arosio, *et al.*, Tree-ring stable isotopes from the European Alps reveal long-term summer drying over the Holocene. *Sci. Adv.* **11**, 4161 (2025).
